# Proliferation dynamics of organotypic vascular endothelium during aging

**DOI:** 10.1038/s44325-026-00148-z

**Published:** 2026-07-25

**Authors:** Yifang Mao, Anna Babin-Ebell Gonçalves, Lorna Rinck, Marlene Hoffarth, Gladys Hofsetz, Miki Kamiyama, Chi-Chung Wu, Junhao Hu, Mahak Singhal

**Affiliations:** 1 AngioRhythms in Health and Disease, European Center for Angioscience (ECAS), Medical Faculty Mannheim, Heidelberg University, Mannheim, Germany; 2 Faculty of Biosciences, Heidelberg University, Heidelberg, Germany; 3 Helmholtz-Institute for Translational AngioCardioScience (HI-TAC) of the Max Delbrück Center for Molecular Medicine in the Helmholtz Association (MDC) at Heidelberg University, Heidelberg, Germany; 4 Ploidy and Organ Function, European Center for Angioscience (ECAS), Medical Faculty Mannheim, Heidelberg University, Mannheim, Germany; 5 Interdisciplinary Research Center on Biology and Chemistry, Shanghai Institute of Organic Chemistry, Chinese Academy of Sciences, Shanghai, China

**Keywords:** Cell biology, Medical research, Physiology

## Abstract

Healthy blood vessels are vital for organismal health. Blood vessel-lining endothelial cells (ECs) can self-replicate to maintain vascular homeostasis, yet the age-related dynamics of EC proliferation remain elusive. Employing cumulative labeling of proliferating ECs, we here present a temporal map of organotypic changes in endothelial proliferation at different stages of mouse life [juvenile (J), 1-month-old; young adult (YA), 4-month-old; old adult (OA), 14-month-old]. Comparative analyses of 12 different organs revealed a tissue-specific pattern of age-related changes in endothelial proliferation capacity. Contrary to the prevailing notion, the majority of analyzed vascular beds retained their proliferative capacity during aging. Intriguingly, lung ECs manifested increased proliferation, whereas adipose, colon, and liver tissues displayed reduced EC proliferation during aging. Together, the data provide a vascular reference framework, highlighting a high degree of organ specificity in the self-renewal capacity of differentiated ECs and uncovering the influence of organismal aging on EC proliferation.

## Introduction

Vascular endothelium forms a systemically disseminated organ that delivers nutrients and facilitates gaseous exchange across our body^1^. Besides these systemic functions, blood vessel-lining endothelial cells (ECs) acquire organ-specific characteristics to critically support and enable physiological organ function^2,3^. In turn, dysfunction of blood vessels disrupts functional homeostasis of an organ and renders tissues vulnerable to diseases, such as cardiovascular diseases, cancer, and inflammation^4–8^. More recently, vascular functional insufficiency was demonstrated to drive age-related decline in organ function^9^, and counteracting vascular deterioration with low-dose systemic VEGF could alleviate multiorgan malfunctioning and prolong overall lifespan. Given the necessity of healthy and resilient vasculature for organismal health, it is surprising that it remains unclear how aging affects the intrinsic ability of differentiated endothelium to proliferate and maintain vascular homeostasis.

During embryonic and early postnatal development, successive processes of vasculogenesis and angiogenesis contribute to the formation and expansion of organotypic vasculature^10^. In adults, differentiated ECs harbor self-renewal capacity whereby tissue-resident ECs can on-demand proliferate to repair damaged vessels and recover from an injury^11,12^. Nevertheless, under steady-state conditions, it was long believed that adult ECs maintain a quiescent cell state, with near-zero to very low cell proliferation^1,3^. Challenging this prevailing notion, recent studies have demonstrated that a substantial fraction of ECs proliferates under physiological homeostatic conditions, with monthly endothelial turnover in mice ranging from ~3% in the aorta, to ~10% in the lung, and ~30% in the liver tissues^13–15^. These compelling findings provide initial evidence of organ-specificity in EC proliferation and hint to a high degree of plasticity in adult ECs.

Building on these recent advances in our knowledge of blood vessels, the present study was designed to answer how EC proliferation changes with organismal aging. To this end, we employed cumulative 5-ethynyl-2′-deoxyuridine (EdU) or 5-bromo-2′-deoxyuridine (BrdU) labeling to monitor EC proliferation at different stages of mouse life [juvenile (J), 1-month-old; young adult (YA), 4-month-old; old adult (OA), 14-month-old]. These temporal analyses uncovered a high degree of variability in EC turnover among vascular beds and revealed an organ-specific pattern of age-related changes in EC proliferative capacity.

## Results

### Establishing cumulative labeling of endothelial and immune cells

**Fig. 1:**
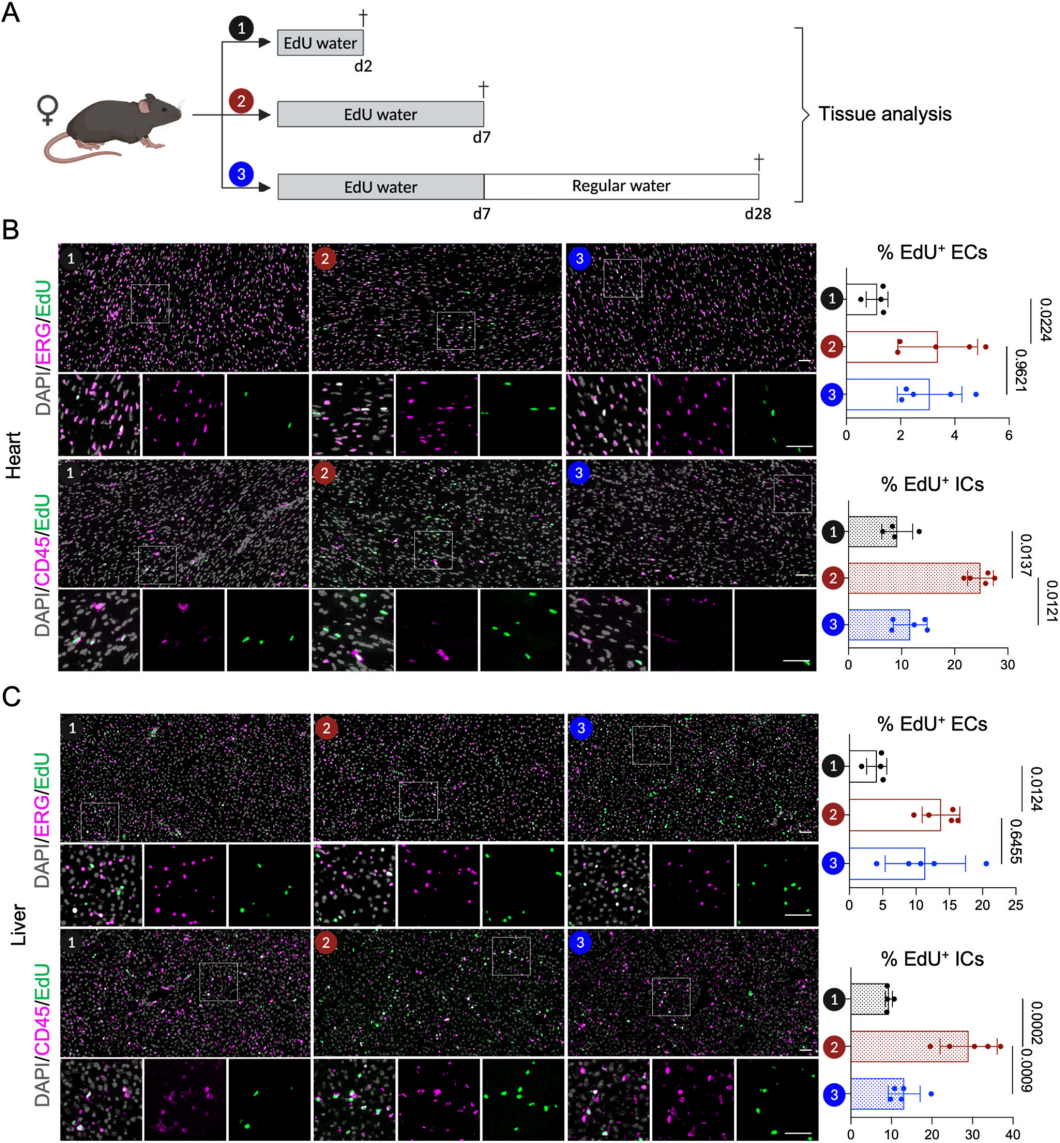
Tracing proliferation kinetics across endothelial and immune cells in mice fed with different durations of EdU-containing drinking water. **A** Schematic illustration of the experimental design. Group 1 mice were fed EdU water for 2 days, and tissues were collected afterward. Group 2 mice were fed EdU water for 7 days, and tissues were collected afterward. Group 3 mice were fed EdU water for 7 days, and regular water for EdU washout for the subsequent 21 days, followed by tissue collection. Representative images and quantitative analysis of EdU+ endothelial and immune cells in heart (**B**) and liver (**C**) tissues of different groups (*n*_*females*_ = 4 in group 1, *n*_*females*_ = 5 each in groups 2 and 3). Data are presented as mean ± SD. *P* values are shown as numerical values computed using one-way ANOVA test. Scale bar: 50 µm.

To investigate age-related changes in EC proliferation, we first characterized in vivo EdU-based cellular labeling assays. To this end, two months old female mice were randomized into three biological groups—group 1 was fed EdU containing water for 2 days; group 2 was fed EdU water for 7 days; and group 3 was fed EdU water for 7 days and afterwards, subjected to a 21 days EdU washout where mice received regular drinking water (Fig. 1A). Heart and liver tissues from these three biological groups were collected and compared for EdU incorporation in both ECs and immune cells (ICs). As expected, we observed an increased EdU labeling of ECs and ICs across both tissues with a longer EdU administration in group 2 when compared to group 1 (Fig. 1B, C). Analyzing tissues from groups 2 and 3, ICs showed a decline in EdU-labeled cells, potentially attributed to the short half-life of various immune subpopulations. In contrast, heart and liver ECs were similarly labeled in tissues from groups 2 and 3, suggesting that ECs retain the EdU labeling even after a 3-week EdU washout period. Interestingly, while we observed a very similar fraction of proliferating ICs between heart and liver tissues, ECs displayed remarkably higher EdU incorporation in the liver when compared to heart tissues, illustrating an organ-specific difference in the proliferation rate of vascular endothelium.

Technically, feeding with EdU water for 7 days labeled approximately 3% of heart ECs with quite high heterogeneity within the biological group (Fig. 1B). To increase the fraction of labeled ECs and potentially reduce intragroup heterogeneity, we opted for longer (28 days) administration of EdU water to mice in subsequent experiments.

### Mapping age-associated changes in EC proliferation capacity

To systematically trace age-related changes in EC proliferation, we compared mice at three different stages of their life: actively growing 1-month-old juvenile (J), fully mature 4-month-old young adult (YA), and early aged 14-month-old adult (OA) mice. We intentionally opted to analyze 14-month-old mice for two reasons. First, previous studies, focusing on the heart^16^, bone^17^, liver^9,18^, and systemic metabolism^9,19^, suggested that an early decline in vascular function drives subsequent age-related physiological decline in organ function. Thus, we questioned whether there is a reduction in endothelial proliferative capacity during early aging in female and male mice and how that varies across different organ vasculatures. Second, 14-month-old mice do not display any overt diseases or organ failure, which may indirectly influence vascular health and impact the proliferation ability of ECs. 1-month-old mice were classified as Juvenile mice, and served as positive controls, as mice rapidly grow during this age with an expanding vasculature. Mice attain physical maturation around 3 months of age; hence, we opted for 4-month-old mice as mature young adults for our analysis.

**Fig. 2:**
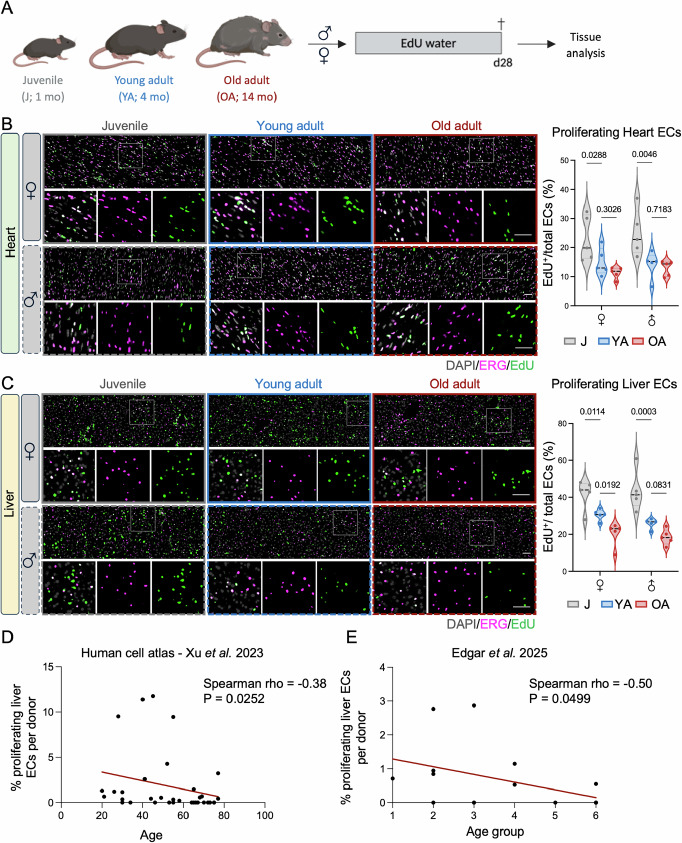
Age-related dynamics of EC proliferation in heart and liver tissues. **A** Schematic illustration of the experimental design. Juvenile (J, 1 mo old), young adult (YA, 4 mo old) and early old adults (OA, 14 mo old) mice were fed EdU water for 28 days. Subsequently, tissues were collected from J (2 mo old), YA (5 mo old) and OA (15 mo old) mice. Representative images and quantitative analysis of EdU+ endothelial cells in heart (**B**) and liver (**C**) tissues of different ages and both sexes (*n*_*males*_ = 5 and *n*_*females*_ = 5 per age group). Quantitative data are presented as violin plots showing interquartile range, with each dot representing a value corresponding to a biological replicate. *P* values are shown as numerical values computed using two-way ANOVA test. Scale bar: 50 µm. Dot plots show the percentage of proliferating liver ECs per donor across ages in the human cell atlas (HCA, **D**) and the pediatric-adult liver (**E**) datasets. For the pediatric-adult dataset, age groups were defined by the authors as 1—child stage (1–4 years), 2—juvenile stage (5–14 years), 3—adolescent stage (15–19 years), 4—young adult stage (20–35 years), 5—prime adult stage (36–55 years), 6—late adult stage ( > 56 years). For human scRNAseq data analysis (**D**, **E**), correlation coefficients (rho) and corresponding p values were computed using Spearman’s rank correlation.

Female and male cohorts of J, YA, and OA mice were fed EdU-containing drinking water for 28 days (Fig. 2A). Subsequently, 12 different tissues (adipose [brown, gonadal, and inguinal white], brain, colon, heart, kidney, liver, lung, muscle, pancreas, and stomach) were collected. We first examined lung and liver tissues for age-related histological changes to ensure that our cohort of 14-month-old mice displayed features of early aging. Here, we observed thickening of interalveolar septa and fibrosis in the lung tissues from OA mice (Supplementary Fig. S1A-B). Similarly, aged liver tissues exhibited hepatocyte ballooning and lipid droplet accumulation (Supplementary Fig. S1C-D). After confirming age-related changes in OA mice, we next computed the EdU-labeled fraction of ECs and ICs in each of the 12 tissues across the three age groups of both female and male mice. Overall, the labeling efficiency of ICs in adult mice ranged from ~10% to ~50%, with the majority of tissues displaying around 30% labeled leukocytes (Table 1). In contrast, ECs exhibited a higher degree of organotypicity in their proliferative capacity, with their labeling ranging from ~1.5% to ~30% in adult mice.

**Table 1:** Age-related dynamics of EC and IC proliferation across different tissues in female and male mice.

	Endothelial cells				Immune cells			
	Female		Male		Female		Male	
	Young adult	Old adult	Young adult	Old adult	Young adult	Old adult	Young adult	Old adult
Liver	30.64 ± 2.97	20.32 ± 6.40	25.94 ± 2.68	18.51 ± 4.18	44.79 ± 5.10	30.40 ± 4.24	37.84 ± 9.94	27.59 ± 7.55
Heart	14.96 ± 4.67	11.35 ± 1.96	14.17 ± 4.58	12.92 ± 2.65	27.59 ± 7.55	37.63 ± 1.95	40.11 ± 5.07	35.09 ± 2.18
Lung	9.60 ± 1.96	13.17 ± 3.04	7.75 ± 0.77	12.34 ± 2.14	22.67 ± 5.81	28.62 ± 4.92	29.07 ± 4.13	21.94 ± 7.59
Kidney	8.08 ± 1.30	8.93 ± 1.76	12.30 ± 0.85	10.31 ± 2.01	18.96 ± 7.10	26.00 ± 2.04	24.51 ± 8.30	24.78 ± 4.93
Stomach	11.07 ± 4.88	7.50 ± 3.40	10.87 ± 5.99	8.30 ± 2.64	29.58 ± 5.77	29.67 ± 8.24	38.75 ± 4.54	39.37 ± 7.89
Pancreas	3.12 ± 0.89	2.58 ± 1.02	3.05 ± 0.86	3.79 ± 2.47	16.05 ± 4.23	15.60 ± 6.32	18.16 ± 5.16	23.34 ± 5.29
Colon	14.63 ± 4.83	4.76 ± 2.17	14.09 ± 3.51	8.99 ± 2.49	29.47 ± 4.82	19.75 ± 7.11	24.67 ± 2.00	22.62 ± 7.21
BAT	2.48 ± 0.84	1.47 ± 0.22	2.24 ± 1.07	2.38 ± 0.72	47.15 ± 7.95	50.12 ± 7.31	42.14 ± 4.35	45.91 ± 8.26
IWAT	16.44 ± 4.86	7.17 ± 3.51	11.56 ± 3.66	7.62 ± 1.43	38.77 ± 4.88	32.42 ± 6.87	41.24 ± 6.71	33.17 ± 5.97
GWAT	11.12 ± 4.29	5.36 ± 1.64	5.96 ± 0.58	6.42 ± 1.57	32.44 ± 3.36	26.43 ± 2.71	32.43 ± 3.90	34.43 ± 3.85
Muscle	4.95 ± 2.11	3.25 ± 1.87	2.49 ± 0.94	2.71 ± 1.91	29.33 ± 8.88	25.36 ± 7.86	23.15 ± 10.48	25.74 ± 5.41
Brain	2.51 ± 0.44	2.70 ± 0.34	2.47 ± 0.18	2.57 ± 0.26	10.21 ± 1.96	10.43 ± 2.27	15.08 ± 3.00	12.05 ± 2.05

All data are presented as mean ± SD.

Comparative analysis across different tissues revealed organ-specific patterns of age-associated changes in cellular proliferation. Surprisingly, EC proliferation did not change with age across multiple tissues, including brain, heart, kidney, muscle, pancreas, and stomach, between YA and OA samples (Fig. 2B, Supplementary Fig. S2A-B, S3A–C, and S4A-B, and Table 1). Unexpectedly, lung tissues from OA mice displayed higher fraction of EdU-labeled ECs as compared to YA lung samples (Supplementary Fig. S4C and Table 1). In contrast, adipose (iWAT), colon and liver tissues exhibited reduced EdU labeling in ECs in OA mice as compared to corresponding tissue types from YA mice (Fig. 2C, Supplementary Fig. S5A–C, S6A, and Table 1). In the context of adipose tissues, we observed sex-specific differences with a more pronounced age-related decline in EC proliferation in females across the analyzed adipose depots (brown, and gonadal and inguinal white) when compared to corresponding male tissues. Likewise, analysis of colon tissues manifested a significant age-associated decline in EC proliferation only in female tissues.

**Fig. 3:**
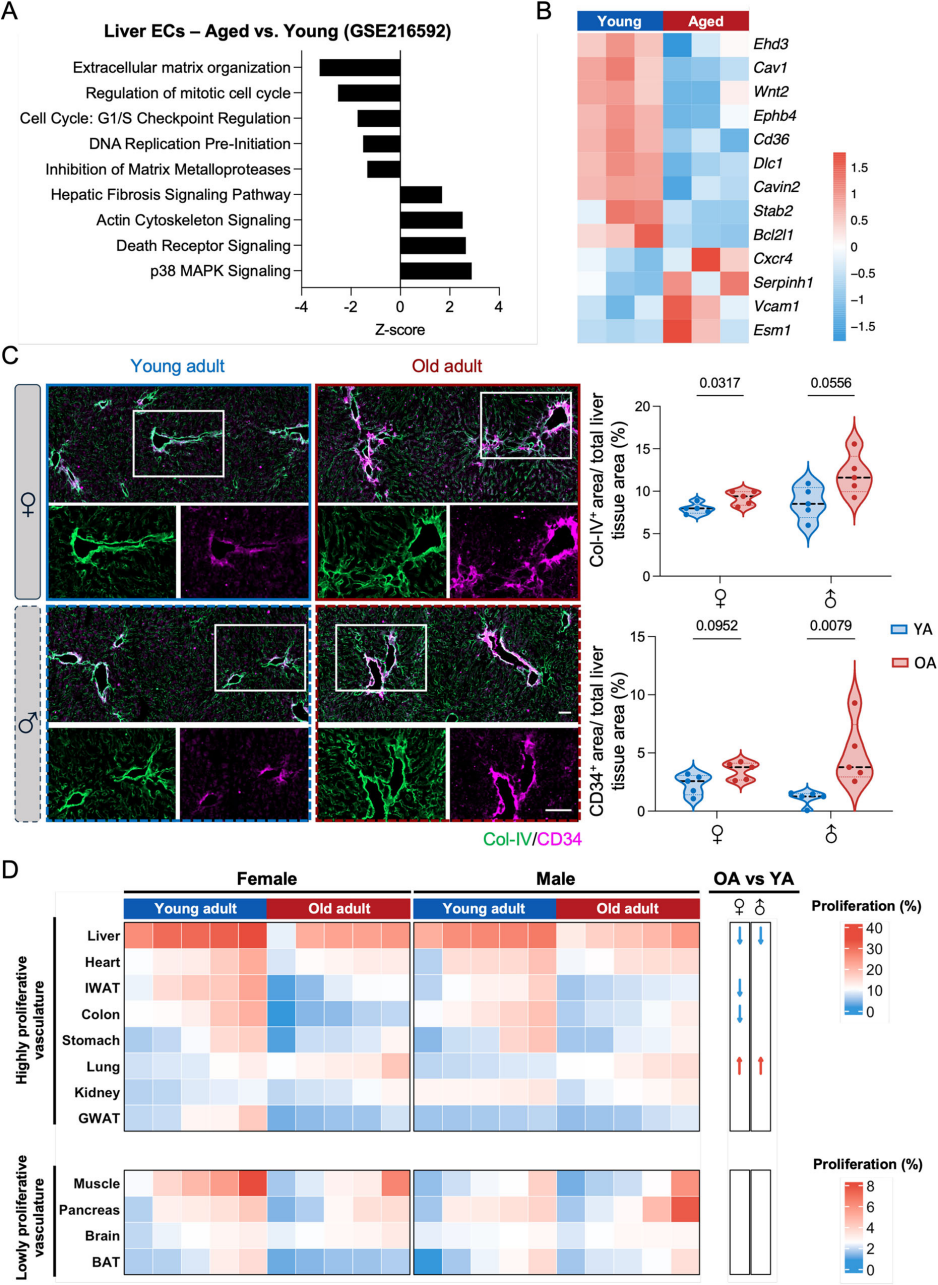
Molecular changes in liver ECs during aging. **A** Aging-associated DEGs in liver ECs were analyzed with Ingenuity Pathway Analysis. Shown are enriched canonical pathways in aged vs. young liver ECs in the GSE216592 dataset. **B** Heatmap comparing the expression of selected DEGs between young and aged liver ECs in the GSE216592 dataset. **C** Representative images and quantitative analysis of CD34 and Col-IV in young and aged livers. (*n*_*males*_ = 5 and *n*_*females*_ = 5 per age group). Quantitative data are presented as violin plots showing interquartile range, with each dot representing a value corresponding to a biological replicate. *P* values are shown as numerical values computed using multiple Mann–Whitney tests. Scale bar: 50 µm. **D** Overview heatmap depiction of EC proliferation (%) across all 12 analyzed organs in young and aged mice (*n*_*males*_ = 5 and *n*_*females*_ = 5 per age group). Organs were stratified based on higher (top) and lower (bottom) average EC proliferation in YA samples.

Liver endothelium marked the highest fraction of EdU-labeled ECs among all analyzed tissues, with ~30% in female and ~26% in male YA samples (Figs. 2C, 3D, and Table 1). Liver ECs showed an age-related decline in their proliferation capacity. This age-associated decline was more pronounced in female mice, potentially as a result of higher proliferation of liver ECs in young female mice when compared to male mice. Furthermore, we analyzed single-cell RNA sequencing (scRNAseq) data from non-diseased human liver tissues from two independent publicly available datasets^20,21^. In line with our findings in mice, we observed a negative correlation between computed percentage of proliferating liver ECs and individual’s age (Fig. 2D, E). These concurrent findings suggest a reduced proliferation capacity of liver ECs in aging mouse and human tissues. Overall, the multiorgan analyses highlight a high degree of organ specificity in age-associated changes in EC proliferation capacity.

To ensure the robustness and reproducibility of our EdU-based findings, we repeated the cumulative labeling experiments in an independent cohort of YA and OA female mice that were fed BrdU-containing water for 2 weeks. Here, we intentionally opted for a 2-week administration to assess whether a shorter cumulative labeling window would reflect a similar pattern of age-related changes in the proliferative capacity of ECs. This would additionally allow to reduce potential toxicities arising from the incorporation of thymidine analogs into the genomic DNA of proliferating cells. Here, analyses of cumulatively labeled BrdU+ ECs revealed no change in heart EC, a decline in liver EC, and an increase in lung EC proliferation in OA compared to YA tissues (Supplementary Fig. S7A–C). Taken together, concurrent observations from EdU and BrdU labeling experiments describe an organotypic pattern of EC proliferation during early aging in mice.

### Age-related changes in lung and liver endothelia drive divergent proliferative responses

Given that lung and liver ECs manifested a divergent pattern of age-related changes in proliferative capacity, we next sought to gain insight into potential molecular mechanisms. To this end, we reanalyzed published transcriptomic data sets, where isolated lung and liver ECs from young and aged mice were profiled^22,23^. Consistent with the cumulative labeling experiments, *Ccnd1* (Cyclin D1) expression was reduced in liver ECs and increased in lung ECs during aging in analyzed transcriptomic data sets (Supplementary Fig. S8A), further illustrating the divergent proliferative response of these vasculatures to aging.

Analyzing differentially expressed genes (DEGs) in aged vs. young liver ECs with Ingenuity Pathway Analysis (IPA), we found an inactivation of cell cycle-related pathways, whereas cell death- and hepatic fibrosis-related pathways were positively enriched (Fig. 3A). Zooming in on the affected genes, we found several genes related to sinusoidal functions of liver ECs, such as *Stab2, Wnt2, Cd36*, and *Cav1*, were downregulated with aging (Fig. 3B). In contrast, capillary genes, such as *Cxcr4, Esm1*, and *Vcam1*, were upregulated in liver ECs during aging. These data suggested an age-related change in liver sinusoidal ECs with a shift from predominant sinusoidal to capillary features. Functionally, we observed a higher abundance of Collagen-IV, an EC-derived collagen, in aged compared to young livers (Fig. 3C). Concurrently, *Serpinh1* (HSP47), a chaperone driving collagen synthesis and maturation, was upregulated in liver ECs during aging (Fig. 3B). Further, CD34 staining was enhanced in the aged liver tissues, suggesting an acquisition of capillary characteristics in the liver vasculature during aging. These age-related structural and functional changes, coupled with reduced expression of cell cycle genes, potentially explain the observed decrease in the proliferation of liver ECs during aging.

In contrast to liver ECs, DEGs from lung ECs correlated with an activation of cell cycle-related pathways, while cell death-related pathways were negatively enriched during aging (Supplementary Fig. S8B). Likewise, upstream regulator analysis illustrated activation of pro-proliferation transcriptional regulators, such as MYC, PI3K, KRAS, and MTOR, while PTEN, TEK (TIE2), and APLNR were negatively enriched as upstream regulators in aged lung ECs (Supplementary Fig. S8C). Intriguingly, HIF-1α was positively enriched as an upstream regulator in the aged lung ECs. In terms of affected DEGs, proliferation-related genes, such as *Akt3, Epas1, Igf2, Kras*, and *Lrg1*, were upregulated, whereas vascular stabilization genes, such as Tie2 (*Tek*) and *Vhl*, were downregulated in lung ECs during aging (Supplementary Fig. S8D). In line with the observed activated HIF-1α signaling, we found downregulation of *Vhl*, a crucial regulator of HIF-1α stability, in aged lung ECs. This is particularly interesting as activated HIF-1α signaling in ECs was previously described to promote angiogenesis^24^, and would potentially explain the observed increase in EC proliferation in aged lung tissues.

## Discussion

Aging acts as a critical risk factor for vascular diseases^25^. In turn, vascular function declines during an individual’s life, and several studies have attributed this functional insufficiency to vascular attrition, age-related reduction in microvessel density^9,16,26–28^. Yet, the primary cause of vascular attrition remains unexplored. The present study aimed to interrogate one of the factors that might contribute to reduced endothelial turnover – the intrinsic ability of ECs to proliferate and self-repopulate. Comparative analysis of 12 different vascular beds between fully matured young adult and early aged mice revealed an organ-specific impact of tissue aging on the proliferative capacity of the vascular endothelium (Fig. 3D).

### Organ-specific EC proliferation code

Analyzing two independent cohorts of female and male mice, we investigated organotypicity in EC proliferation. Brain vasculature, with the strongest barrier function, displayed the lowest EC proliferation with merely 2.5% ECs undergoing DNA replication during the tracing period of 4 weeks. Conversely, liver vasculature, with fenestrated endothelium largely devoid of basement membrane, showed the highest proliferation with nearly 30% liver ECs incorporating EdU. In the context of adipose tissues, white adipose depots (inguinal and gonadal) exhibited substantially higher EC proliferation when compared to brown adipose tissues. In line with our increasing understanding of functional heterogeneity among different vascular endothelia and growing appreciation of organ-specific characteristics of capillary ECs^29–31^, it is not too far-fetched to hypothesize that different organ vasculatures have different cellular turnover rates. Our findings would definitely support that hypothesis, as different organ ECs illustrate varying degrees of cellular proliferation.

### Age-associated changes in EC proliferation

Unexpectedly, a large subset of organs did not demonstrate any change in EC proliferation ability during the early aging process. Among endothelia with age-related alterations, liver, colon, and adipose (iWAT) tissues showed a reduction, whereas the lung displayed an increase in EdU-labeled ECs. It is noteworthy that lung and liver vasculatures are perhaps two of the most biophysically challenged vasculatures under steady-state conditions. While the lung expands with every breath, the liver undergoes daily oscillations in mass, with hepatocytes growing nearly 20% after the feeding phase^32^. These biophysical changes in lung and liver tissues impose stretch on the respective vasculature. Yet, it is counterintuitive that these two organ vasculatures manifested opposite age-related changes in their proliferation capacity. In the context of the liver, analysis of bulk transcriptomics data from aged ECs suggested a loss of sinusoidal characteristics and acquisition of capillary features. This was also reflected in increased CD34 and Collagen-IV staining in aged liver tissues compared to young livers. These findings align well with previous reports of altered liver vasculature with swelling and pseudo-capillarization of sinusoidal ECs^18,23,33^. It was notable that livers from female mice demonstrated substantially higher EC proliferation when compared to male liver tissues at corresponding ages (YA and OA), potentially contributing to sex-specific response to different liver pathological challenges^34^. In the context of the lung, transcriptomic analyses revealed an enrichment of cell cycle-related gene sets. Intriguingly, in silico analysis revealed an activation of HIF-1α signaling in aged lung ECs, potentially driving the enhanced proliferation. The activated HIF-1α signaling in aged lungs might be attributable to age-associated fibrosis, increased interalveolar septa thickness, and inflammation^35^. While the present study provides initial mechanistic hypothesis for these diverging responses of lung and liver vasculatures, future studies will need to comprehensively investigate the underlying cellular and molecular crosstalk in these tissue microenvironments and assess the impact of organismal aging.

### Nascent DNA versus genetic labeling

The present study employed EdU- and BrdU-based labeling of nascent DNA to track cells that undergo DNA replication. Labeling nascent DNA enabled us to compare cellular proliferation kinetics across different ages of mice. Pursuing similar age-related tracing experiments with genetic labeling of a specific cell type are often logistically challenging and require aging of cohorts of transgenic mice. We circumvented this caveat with cumulative labeling of ECs and ICs in commercially acquired aged wild-type mice. Nevertheless, to ensure robustness and compatibility of our findings, we compared our results with a recently published study where the authors traced EC proliferation with genetic labeling strategies over 4 weeks in healthy young adult mice. Consistently, our nascent DNA approach displayed comparable EC labeling efficiency to the reported genetic labeling for the liver, lung and heart ECs in young adult mice^14^. Furthermore, we analyzed publicly available human single-cell RNA sequencing (scRNAseq) datasets from non-diseased individuals and assessed whether the age-related changes in EC proliferation occur similarly between mice and humans. This turned out to be challenging as (i) most of the existing whole organ scRNAseq datasets had very few high-quality ECs, and (ii) these are primarily snapshot analysis which rarely capture proliferating ECs. Nevertheless, for the liver, where we had observed highest EC proliferation, reanalysis of two independent human liver scRNAseq data sets showed an age-associated reduction in liver EC proliferation. These findings are concurrent with our wet lab experiments in mice. Lastly, we believe opting for longer EdU administration in drinking water allowed us to robustly label proliferating ECs across different organs.

Taken together, the current study presents proliferation dynamics of organotypic vascular endothelium and describes sex- and age-associated alterations in the vascular proliferation kinetics.

## Methods

### Mice

C57BL/6JRj mice of mentioned age and sex were purchased from Janvier Laboratories. The C57BL/6JRj strain is a rederivation of the original C57BL/6J strain from the Jax Laboratory. All mice were housed on a 12 h light/12 h dark cycle with free access to food and drinking water in specific pathogen-free animal facilities. All animal experiments were approved by the governmental (G24-25 from Regierungspräsidium Karlsruhe, Germany) and Institutional (I25-10 to M.S. and ECSIOC_2025-51 to J.H.) Animal Care and Use Committees. All experiments were performed in accordance with the respective institutional guidelines for the care and use of laboratory animals.

### Study design

The objective of this study was to investigate the age-associated changes in the endothelial proliferation capacity. This was a hypothesis-driven study with unknown “effect magnitude.” Therefore, statistical power was not computed before the experiments. The used sample sizes were estimated on the basis of the previous experiences. Individual mice served as biological replicates within an in vivo experiment. Mice were acquired commercially in pre-defined cohorts for each age group and were acclimatized to local housing conditions for two weeks before initiating an in vivo experiment. For immunofluorescence image quantitation, image regions were excluded if artifacts were observed during automated signal thresholding. Scientists were blinded while performing image analyses.

### In vivo EdU administration in drinking water

The synthetic deoxyribonucleoside analog 5-ethynyl-2’-deoxyuridine (EdU, Carbosynth, NE08701) was administered to mice in drinking water at 0.2 mg/ml for the mentioned time. EdU containing water was replaced every 2–3 days.

### In vivo BrdU administration in drinking water

5-Bromo-2’-deoxyuridine (BrdU, Sigma-Aldrich, B2850.5) was administered to mice in drinking water with 2% sucrose at 0.8 mg/ml for 2 weeks. BrdU containing water was replaced every 2–3 days.

### Tissue collection and sectioning

At the experimental endpoint, mice were euthanized by cervical dislocation. Mouse tissues (adipose [brown, gonadal, and inguinal white], brain, colon, heart, kidney, liver, lung, muscle, pancreas, and stomach) were freshly collected. Brain, brown adipose, heart, liver, and kidney tissues were immediately placed into optimal cutting temperature (OCT) compound, frozen on dry ice, and stored subsequently at –80 °C. Stomach tissues were rinsed in PBS and washed thoroughly, then the corpus of the stomach was separated from other regions and frozen in OCT on dry ice. The Swiss roll embedding method was used to collect colon tissues before freezing on dry ice. All the above samples were sectioned with a Leica CM3050 S cryostat. Lung, pancreas, inguinal, and gonadal white adipose tissues were dissected, collected and fixed in ice-cold 4% paraformaldehyde (PFA) overnight. Samples were embedded in a paraffin block and sliced into 4-µm-thick sections using a Leica HistoCore BIOCUT rotary microtome.

### Tissue staining

For immunofluorescence staining on cryo sections, sections were fixed with 4% PFA (Carl Roth, Cat #0335.1) for 15 min at room temperature (RT). After blocking and permeabilization in 5% Fetal Bovine Serum (FBS) and 0.5% TritonX-100 (VWR, Cat#0694-1 L) at RT for 45 min, samples were incubated with rabbit anti-mouse ERG (Abcam, Cat#ab92513; 1:500) or rat anti-mouse CD45 (BioLegend, Cat#103102; 1:400) antibody diluted in blocking buffer overnight at 4 °C. Following three times washes (each for 5 min) in TBST solution, sections were then incubated with a mix composed of Alexa Fluor 647-conjugated donkey anti-rabbit (Thermo Fisher Scientific, Cat#A-31573, 1:500) or AF647 donkey anti-rat (Thermo Fisher Scientific, Cat# A-78947, 1:500) and Hoechst (Invitrogen, Cat #H1399, 1:2000) for 1 h at RT.

For Col-IV and CD34 immunofluorescence staining, liver tissue cryo sections were fixed with methanol (Häberle Lab, Cat# HC112161000) at –20 °C. After blocking in 5% FBS at RT for 45 min, liver sections were incubated with goat anti-mouse Col-IV (R&D Systems, Cat#NBP1-26549, 1:400) and rat anti-mouse CD34 (Invitrogen, Cat#14-0341-82, 1:200) antibodies diluted in blocking buffer overnight at 4 °C. After three washes (5 min each) in TBST solution, sections were incubated with AF647-conjugated donkey anti-rat and AF488-conjugated donkey anti-goat (Thermo Fisher Scientific, Cat# A-11055, 1:500) and Hoechst for 1 h at RT.

For detection of EdU^+^ cells, the slides were incubated in Click-iT reaction cocktail (Baseclick, Cat#BCK-EdUPro488IM100) for 30 min at RT in dark conditions. The slides were then washed with TBST for 5 min at least 3 times.

For detection of BrdU⁺ cells, cryo sections fixed with 4% PFA were incubated in 2 M HCl (Merck, Cat# 258148-500 ML) for 45 min at 37 °C. Sections were subsequently neutralized with 0.1 M sodium borate (Sigma-Aldrich, Cat# S9640-500G) for 10 min at RT. After blocking and permeabilization with 5% bovine serum albumin (BSA; GERBU, Cat#1063-100 g) and 0.5% Triton X-100, sections were incubated overnight at 4 °C with rat anti-mouse BrdU antibody (Abcam, Cat#ab6326; 1:500) diluted in blocking buffer. After three washes in TBST (5 min each), sections were incubated with AF488-conjugated donkey anti-rat secondary antibody (Thermo Fisher Scientific, Cat# A-21208; 1:500) and Hoechst (Invitrogen, Cat# H1399; 1:2000) for 1 h at RT.

For tissue sections generated from paraffin-embedded tissue blocks, tissue sections were deparaffinized in xylene twice (each for 5 min) at RT. After dehydration through graded ethanol solutions and rinsing in cold water, sections were incubated in antigen retrieval solution, citrate pH6 (Agilent Technologies, Cat#S169984-2) at 95 °C for 20 min and washed thoroughly in PBS. Later, sections were processed for immunostaining, as described above.

Regarding to the Oil O Red staining, cryo sections (8 µm) were air-dried at RT for 45 min and fixed in 10% Neutral Buffered Formalin (NBF, Sigma-Aldrich, Cat#HT501128-4L) for 10 min. After rinsing, sections were equilibrated in 60% isopropanol for 5 min and then stained with freshly prepared and filtered Oil Red O working solution for 20 min. Nuclei were counterstained with filtered Hemalum solution for 3 min, and sections were washed under running tap water.

The Hematoxylin & Eosin (H&E) staining and Masson Goldner staining were done on paraffin-embedded slides by the General Core Equipment unit of the Medical Faculty Mannheim of Heidelberg University according to their standard operating procedures. All stained slides were scanned using an Axioscan 7 system.

### Quantification of imaging datasets

Quantification of cell numbers, such ERG+ endothelial cells and CD45+ immune cells, were conducted with Fiji based on the ERG/EdU and CD45/EdU stained images. Cells that were double positive with ERG and EdU were considered as proliferating endothelial cells. Cells that were double positive with CD45 and EdU were considered as proliferating immune cells.

The stained area of CD34 and Col-IV were quantified using a uniform threshold after channel splitting, and the percentage of positive staining was calculated as the ratio of the stained area to the total tissue area.

### Proliferation index analysis

To evaluate proliferative activity in publicly available human liver endothelial single-cell RNA-sequencing (scRNAseq) datasets, we obtained preprocessed data in h5ad format from the CELLxGENE platform (https://cellxgene.cziscience.com/collections/ff69f0ee-fef6-4895-9f48-6c64a68c8289, https://cellxgene.cziscience.com/collections/854c0855-23ad-4362-8b77-6b1639e7a9fc)^20,21^. Minimal quality control was applied by excluding cells with fewer than 1000 detected counts and genes expressed in fewer than 10 cells. The data were subsequently normalized, log-transformed, and scaled using Scanpy (*v1.11.0*)^36^. To quantify proliferation, we computed a G2/M score for each cell in the dataset by using the G2/M gene set from the hallmark gene set collection (HALLMARK_G2M_CHECKPOINT, M5901)^37^. Cells were classified as proliferating or non-proliferating according to their G2/M score, using dynamically adjusted thresholds that were optimized to yield an overall proliferation fraction of 1–2%. Finally, the proportion of proliferating cells was then calculated for each donor, and the relationship between donor age and proliferation rate was assessed using Spearman’s rank correlation coefficient from the SciPy package (v1.15.2)^38^.

### Processing of public bulk transcriptomic datasets of liver and lung ECs

The publicly available bulk transcriptomic datasets of liver and lung ECs were accessed through Gene Expression Omnibus (GEO) under the accession numbers GSE216592 and GSE181508 respectively. The corresponding Excel-files were downloaded, and the raw counts were extracted for young and aged EC samples (liver ECs: n_young_ = 3, n_aged_ = 3; Lung ECs: n_young_ = 5, n_aged_ = 3). In the case of lung ECs, only the non-treated samples (sham group) were used for further analyses. The count data were loaded into an AnnData object (*v.0.12.4*) and preprocessed with the decoupler framework (*v.2.1.4*)^39^ with default parameters and normalized to transcripts per million (TPM). Differential expression analysis was performed for each dataset using the Python implementation of DESeq2 (pyDESeq2, *v.0.5.3*)^40,41^. Differentially expressed genes (DEGs) between age groups were identified based on a significance threshold of p value < 0.05 and used as input for downstream functional analysis with IPA (QIAGEN Inc.).

### Statistical analysis

Statistical analysis was performed with GraphPad Prism 9.0. *P* value < 0.05 is considered statistically significant. All graphs show each biological replicate. The corresponding statistical test is mentioned in the respective figure legend.

## Supplementary information

Mao-et-al-Suppl-Materials

## Data Availability

Data availability: All data generated in the study can be requested from the corresponding author. No new transcriptomic datasets were generated within this study.

## Code availability

No new codes were generated in the study.
