# Supplementary material for: Proliferation dynamics of organotypic vascular endothelium during aging
Source: NPJ Cardiovasc Health. 2026 Jul 25;3:46. doi: 10.1038/s44325-026-00148-z (PMC13415563; doi:10.1038/s44325-026-00148-z)
Supplement: Supplementary file 1 — Mao-et-al-Suppl-Materials [file 44325_2026_148_MOESM1_ESM.pdf]

Proliferation dynamics of organotypic vascular endothelium during aging

Yifang Mao[1,#], Anna Babin-Ebell Gonçalves[1,2,#], Lorna Rinck[1], Marlene Hoffarth[1,3],

Gladys Hofsetz[3], Miki Kamiyama[1,3], Chi-Chung Wu[3,4], Junhao Hu[5], Mahak Singhal[1,3,*]

(Supplementary Figures S1-S8)

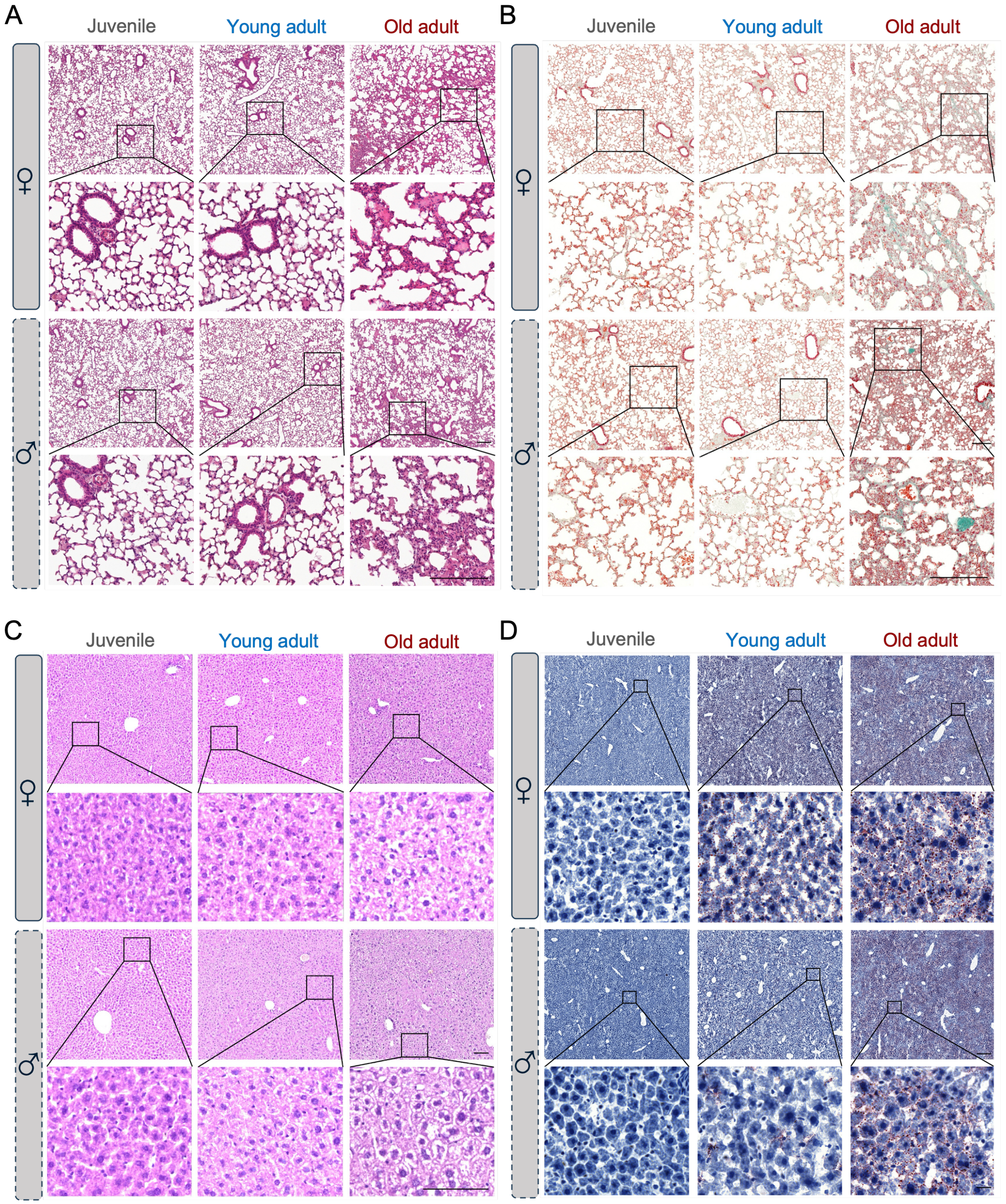

Figure S1: Age-related histological characterization of lung and liver tissues in female and male mice. (A) Representative images of H&E staining on lung tissues. Scale bar: 200 µm. **(B)** Representative images of Masson Goldner staining on lung tissues. Scale bar: 200 µm. **(C)** Representative images of H&E staining on liver tissues. Scale bar: 200 µm. **(D)** Representative images of Oil O Red staining on liver tissues. Scale bar: (overview) 200 µm; (zoom-in) 20 µm.

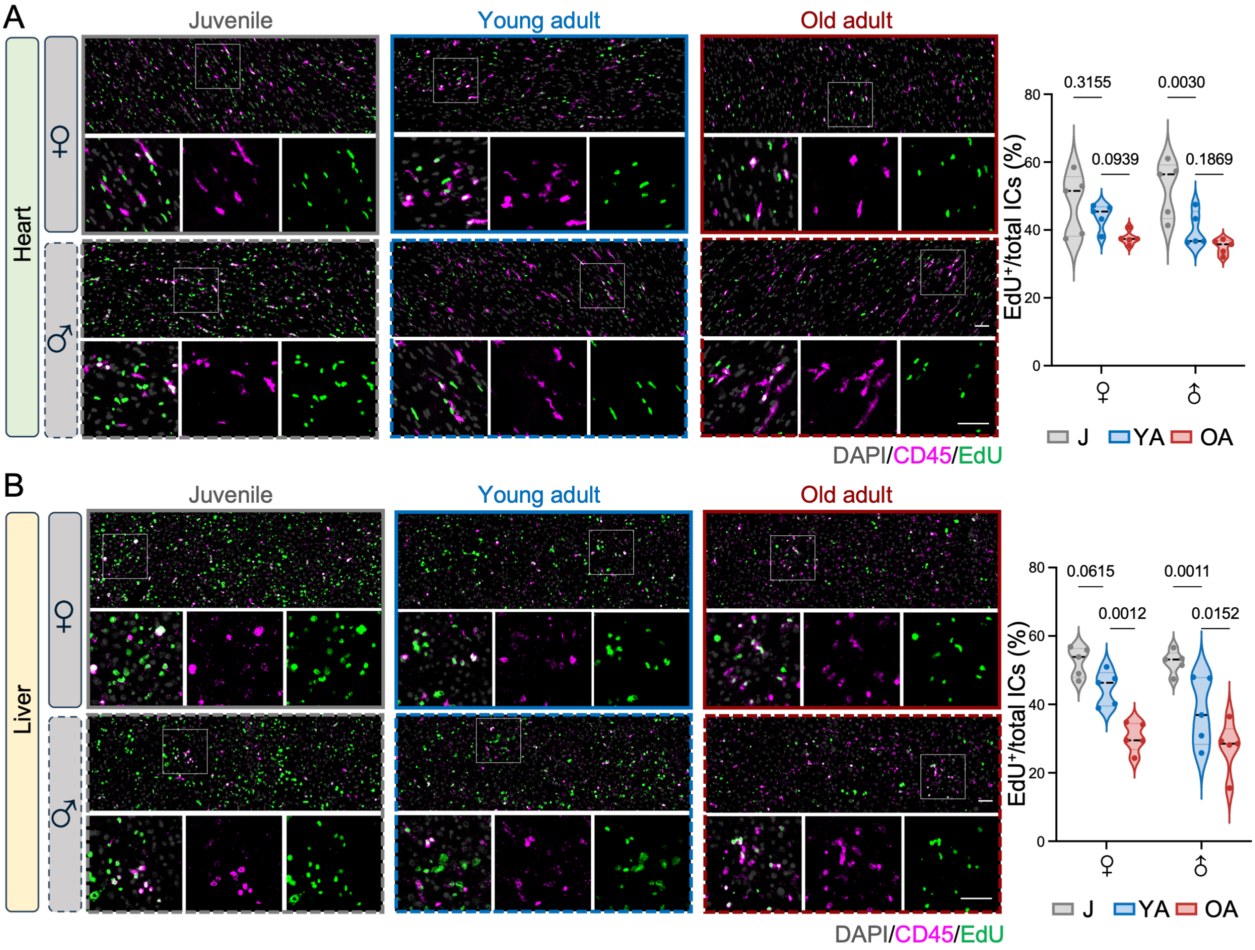

Figure S2. Immune cell proliferation in heart and liver tissues during aging. (A,B) Representative images and quantitative analysis of EdU+ immune cells in heart (**A**) and liver (**B**) tissues from mice of different ages and sexes (n_{males} = 5 and $n_{females}$ = 5 per age group). Quantitative data are presented as violin plots showing interquartile range, with each dot representing a value corresponding to a biological replicate. *P*-values are shown as numerical values computed using two-way ANOVA test. Scale bar: 50 µm.

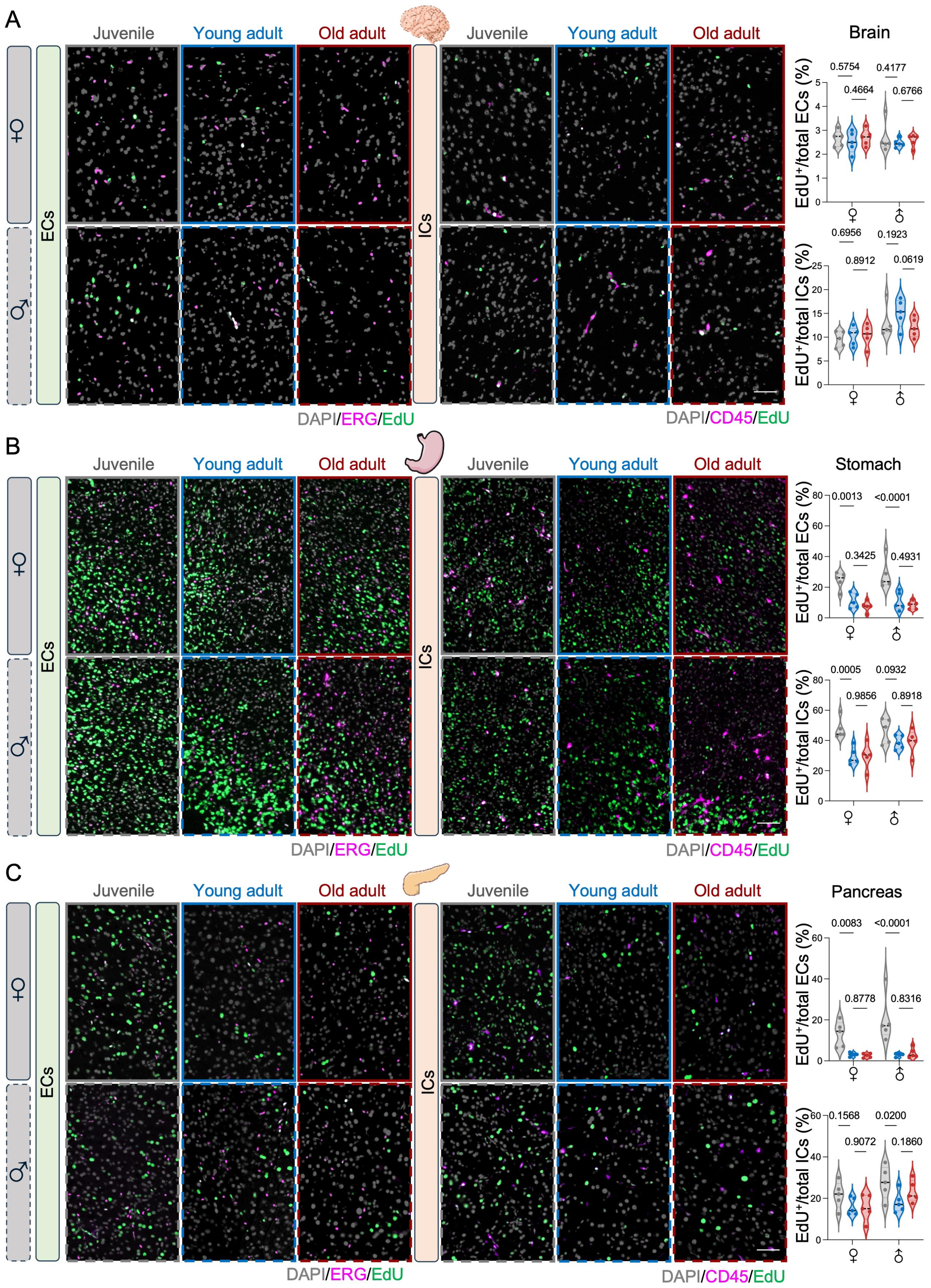
A
Juvenile
Young adult
Old adult
ECs
ICs
♀
♂
DAPI/ERG/EdU
DAPI/CD45/EdU
Brain
EdU+/total ECs (%)
EdU+/total ICs (%)
0.5754
0.4664
0.4177
0.6766
0.6956
0.8912
0.1923
0.0619
B
Stomach
0.0013
0.3425
<0.0001
0.4931
0.0005
0.9856
0.0932
0.8918
C
Pancreas
0.0083
0.8778
<0.0001
0.8316
0.1568
0.9072
0.0200
0.1860

Figure S3. Age-related dynamics of EC and IC proliferation in brain, stomach, and pancreas tissues. (A-C) Representative images and quantitative analysis of EdU+ ECs and ICs in brain (**A**), stomach (**B**), and pancreas (**C**) tissues from mice of different ages and sexes (n_{males} = 5 and $n_{females}$ = 5 per age group). Quantitative data are presented as violin plots showing interquartile range, with each dot representing a value corresponding to a biological replicate. *P*-values are shown as numerical values computed using two-way ANOVA test. Scale bar: 50 µm.

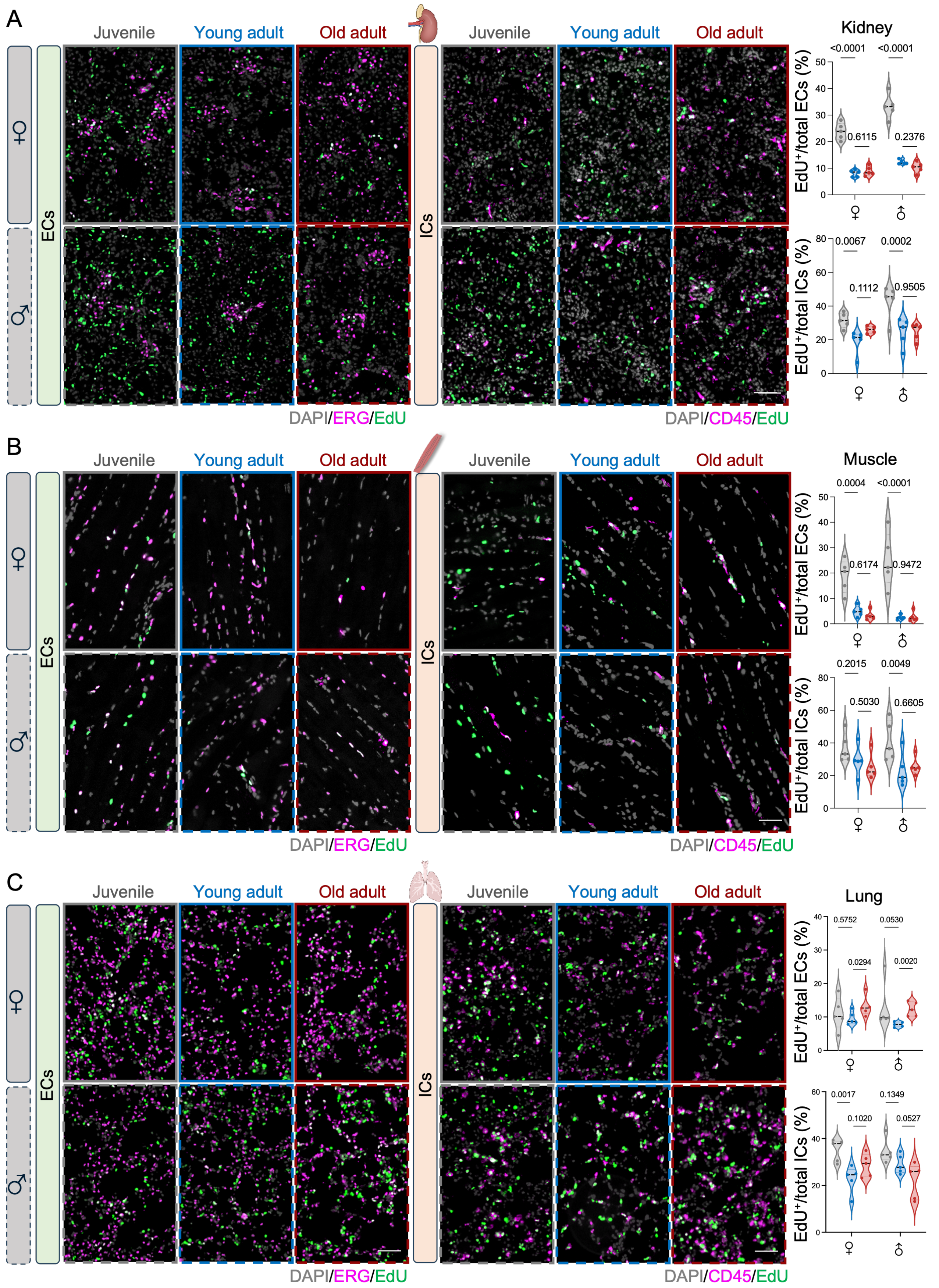
A
Juvenile
Young adult
Old adult
ECs
ICs
Kidney
EdU⁺/total ECs (%)
<0.0001
<0.0001
0.6115
0.2376
EdU⁺/total ICs (%)
0.0067
0.0002
0.1112
0.9505
DAPI/ERG/EdU
DAPI/CD45/EdU
B
Juvenile
Young adult
Old adult
ECs
ICs
Muscle
EdU⁺/total ECs (%)
0.0004
<0.0001
0.6174
0.9472
EdU⁺/total ICs (%)
0.2015
0.0049
0.5030
0.6605
DAPI/ERG/EdU
DAPI/CD45/EdU
C
Juvenile
Young adult
Old adult
ECs
ICs
Lung
EdU⁺/total ECs (%)
0.5752
0.0530
0.0294
0.0020
EdU⁺/total ICs (%)
0.0017
0.1349
0.1020
0.0527
DAPI/ERG/EdU
DAPI/CD45/EdU

Figure S4. Age-related dynamics of EC and IC proliferation in kidney, muscle, and lung tissues. (A-C) Representative images and quantitative analysis of EdU+ ECs and ICs in kidney (**A**), muscle (**B**), and lung (**C**) tissues from mice of different ages and sexes (n_{males} = 5 and $n_{females}$ = 5 per age group). Quantitative data are presented as violin plots showing interquartile range, with each dot representing a value corresponding to a biological replicate. *P*-values are shown as numerical values computed using two-way ANOVA test. Scale bar: 50 µm.

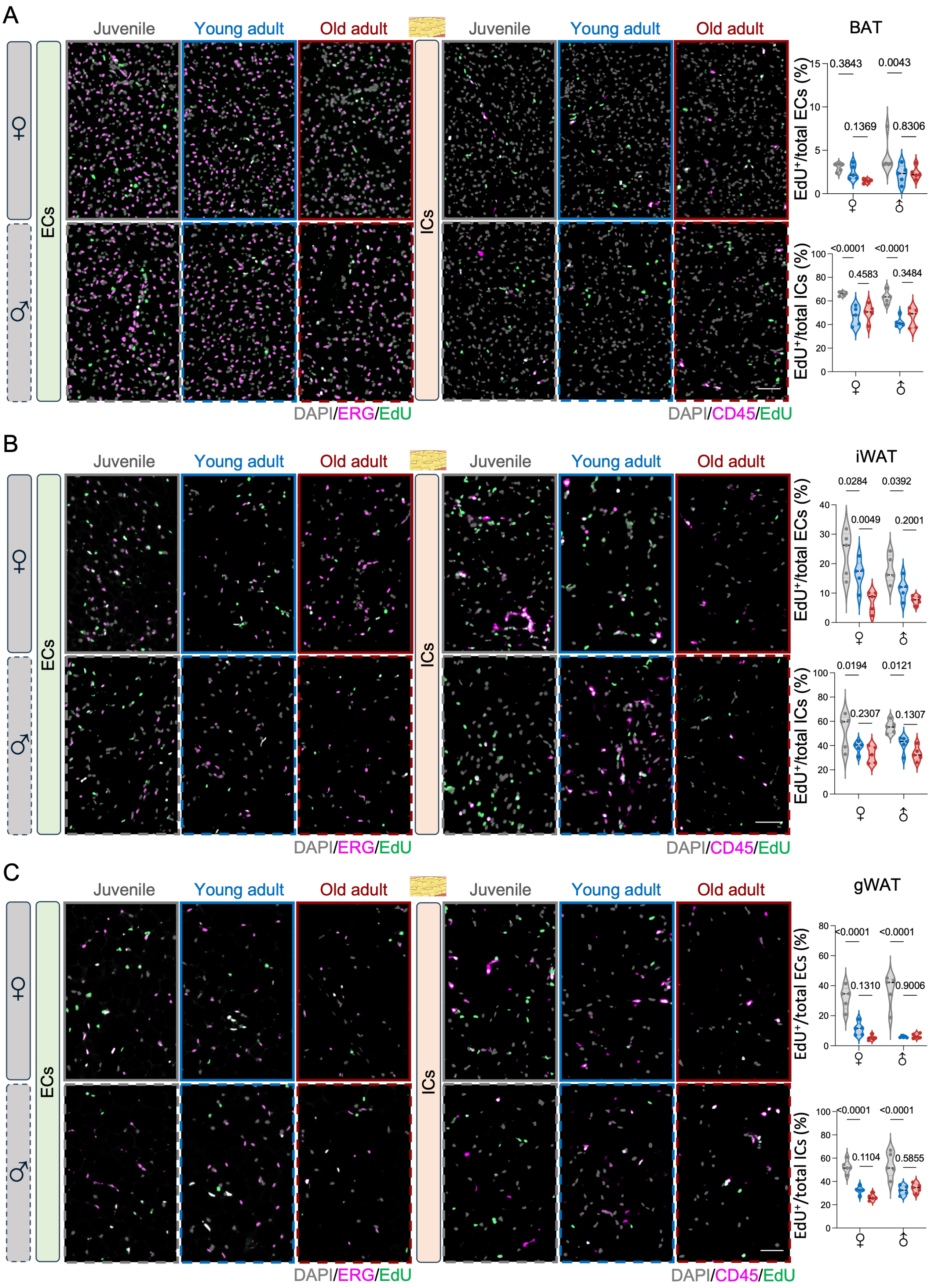
A
Juvenile
Young adult
Old adult
ECs
ICs
♀
♂
DAPI/ERG/EdU
DAPI/CD45/EdU
BAT
EdU+/total ECs (%)
0.3843
0.0043
0.1369
0.8306
EdU+/total ICs (%)
<0.0001
<0.0001
0.4583
0.3484
B
iWAT
0.0284
0.0392
0.0049
0.2001
0.0194
0.0121
0.2307
0.1307
C
gWAT
<0.0001
<0.0001
0.1310
0.9006
<0.0001
<0.0001
0.1104
0.5855

Figure S5. Age-related dynamics of EC and IC proliferation in different adipose depots. (A-C) Representative images and quantitative analysis of EdU+ ECs and ICs in brown (BAT, **A**), inguinal white (iWAT, **B**), and gonadal white (gWAT, **C**) tissues from mice of different ages and sexes (n_{males} = 5 and $n_{females}$ = 5 per age group). Quantitative data are presented as violin plots showing interquartile range, with each dot representing a value corresponding to a biological replicate. *P*-values are shown as numerical values computed using two-way ANOVA test. Scale bar: 50 µm.

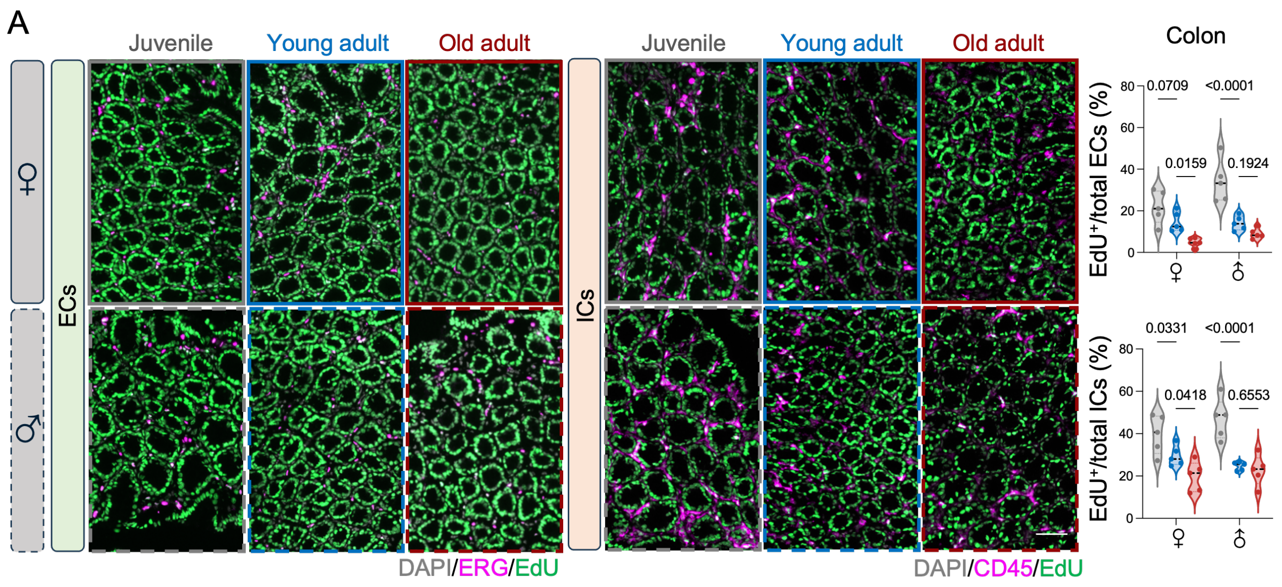

Figure S6. Age-related dynamics of EC and IC proliferation in colon tissues. (A) Representative images and quantitative analysis of EdU+ ECs and ICs in colon tissues from mice of different ages and sexes (n_{males} = 5 and $n_{females}$ = 5 per age group). Quantitative data are presented as violin plots showing interquartile range, with each dot representing a value corresponding to a biological replicate. *P*-values are shown as numerical values computed using two-way ANOVA test. Scale bar: 50 µm.

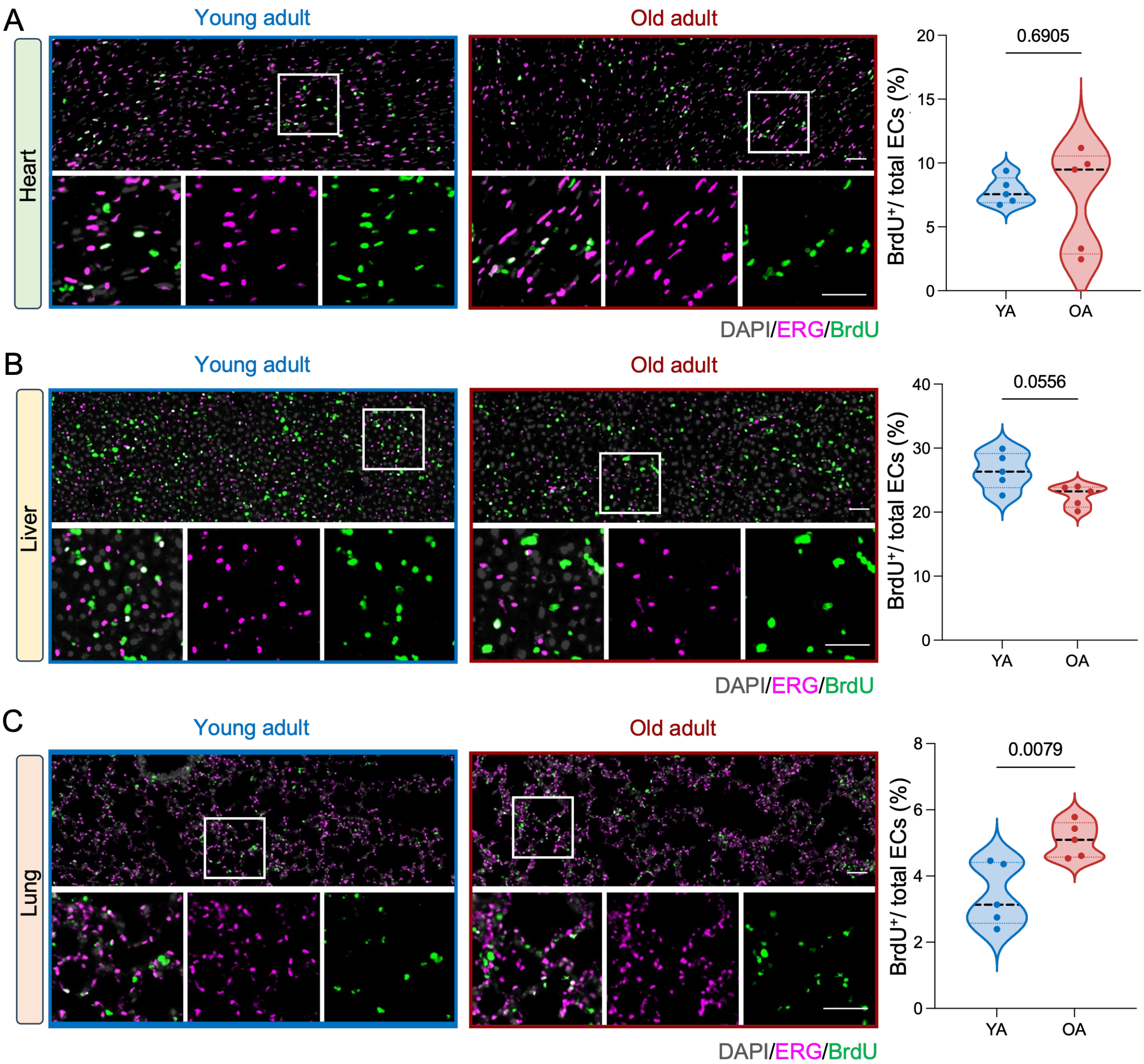

Figure S7. Age-related dynamics of BrdU-based EC proliferation in heart, liver, and lung tissues in female mice. (A-C) Representative images and quantitative analysis of BrdU+ ECs in heart (**A**), liver (**B**), and lung (**C**) tissues from female mice of different ages ($n_{females}$ = 5 per age group). Quantitative data are presented as violin plots showing interquartile range, with each dot representing a value corresponding to a biological replicate. *P*-values are shown as numerical values computed using Mann-Whitney U test. Scale bar: 50 µm.

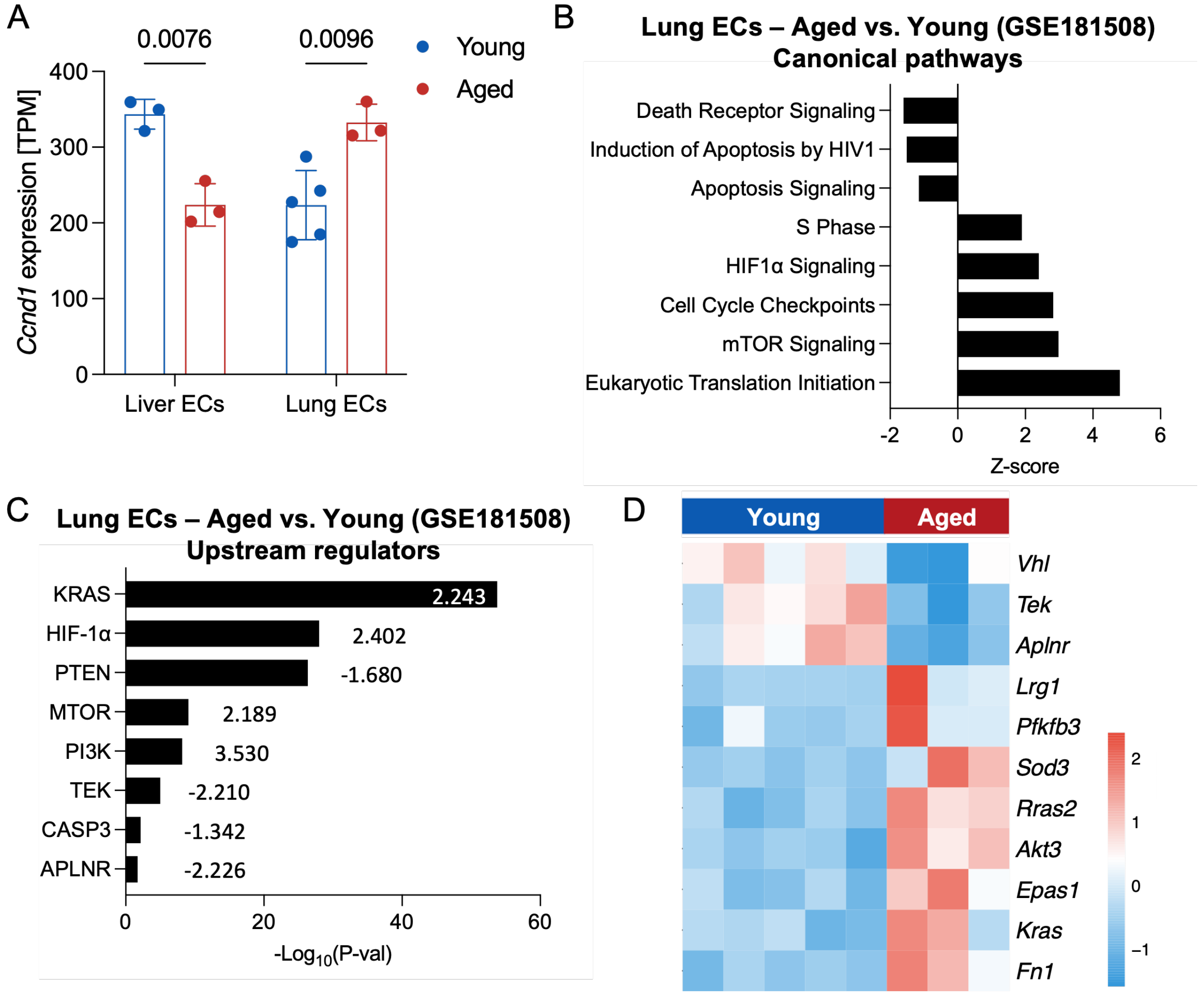

Figure S8. Molecular changes in lung ECs during aging. (A) *Ccnd1* expression in liver and lung ECs isolated from young and aged mice, reanalyzed from publicly available bulk transcriptomic datasets (GSE216592 – liver ECs; GSE181508 – lung ECs). Data are presented as mean ± SD. *P*-values are shown as numerical values computed using multiple Mann-Whitney tests. TPM: Transcripts Per Million reads. **(B,C)** Aging-associated DEGs in lung ECs were analyzed with Ingenuity Pathway Analysis. Shown are enriched canonical pathways (**B**) and upstream regulators (**C**) in aged vs. young lung ECs in the GSE181508 dataset. In panel C, numerical values reflect the corresponding z-score. **(D)** Heatmap comparing the expression of selected DEGs between young and aged lung ECs in the GSE181508 dataset.
